# PD‐L1/TIGIT bispecific antibody showed survival advantage in animal model

**DOI:** 10.1002/ctm2.754

**Published:** 2022-05-06

**Authors:** Songlin Mu, Zhijuan Liang, Yongmei Wang, Wendi Chu, Yi‐Li Chen, Qi Wang, Guifeng Wang, Chunhe Wang

**Affiliations:** ^1^ Biotherapeutics Discovery Research Center Shanghai Institute of Materia Medica Chinese Academy of Sciences Shanghai China; ^2^ University of Chinese Academy of Sciences Beijing China; ^3^ Dartsbio Pharmaceuticals, Ltd. Zhongshan China; ^4^ Shanghai Mabstone Biotechnologies, Ltd. Shanghai China

Dear Editor,

Although PD‐1 and PD‐L1 (programmed cell death‐1/ligand 1) antibody therapies have provided persistent benefits in various cancers, their clinical applications have been limited by relatively low response rates and the occurrence of drug resistance.^1^ Thus, they have been extensively investigated in combination with a variety of antitumor therapies, such as inhibitors to other immune checkpoints in combinational or bispecific antibody (BsAb) therapeutical strategies, to improve the response rates or efficacies in the clinic.^2^

TIGIT (T‐cell immunoreceptor with immunoglobulin and ITIM domains) is an inhibitory T‐cell immunoreceptor discovered in 2009,^3^ which is expressed mainly on regulatory T cells (Tregs), memory T‐cells, and NK cells. Combination therapies that block both PD‐L1 and TIGIT pathways have superior clinical benefits to PD‐L1 monotherapy.^4^ BsAbs simultaneously targeting both pathways are also entering clinical trials (NCT05102214), but whether BsAbs are superior to combinational approaches has not been elucidated yet.

We reported here a novel bispecific anti‐PD‐L1/TIGIT antibody with potent effects in enhancing human IL‐2 production by primary human T cells in vitro and in increasing the overall survival in a transgenic mouse model in vivo. More importantly, we compared the tetravalent bispecific anti‐PD‐L1/TIGIT antibody to mAb and combination therapies head‐to‐head systematically, and our data supported the the advantage of taking bispecific approach over mAb and combination therapies experimentally.

First, hybridomas were established^5^ and humanization was carried out through "framework shuffling^6^, ^7^ on both anti‐PD‐L1 and anti‐TIGIT antibodies. Both chimeric (anti‐PD‐L1‐8A1 and anti‐TIGIT‐31C3) and humanized (anti‐PD‐L1‐#77, anti‐TIGIT‐T26 and T87) antibodies showed binding and blocking capabilities comparable to corresponding positive controls (Figure 1A–D), i.e., atezolizumab (Tencentriq) for anti‐PD‐L1 and tiragolumab for anti‐TIGIT. Affinities were determined by biolayer interferometry (BLI) on OCTET and similar KD values were obtained (Figure S1). For further preclinical safety evaluation and in vivo efficacy studies, cross‐reactive binding assays were evaluated, with high binding abilities to cynomolgus monkey antigen around 0.07 nM (anti‐PD‐L1) and 0.25–2.0 nM (anti‐TIGIT), and no cross‐reactivity to mouse antigen (Figure S2).

**FIGURE 1:**
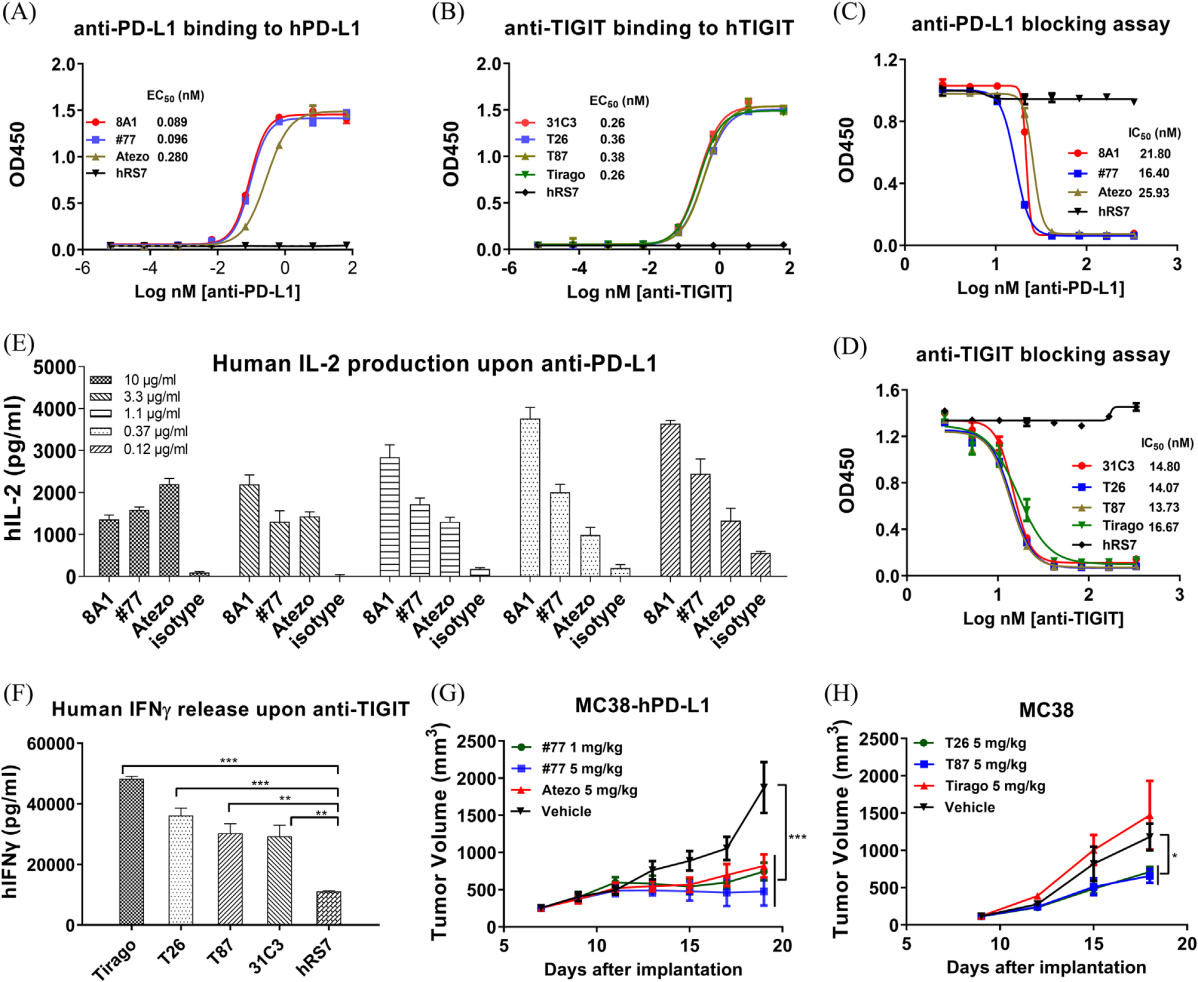
Characterization of anti‐PD‐L1 and anti‐TIGIT mAbs. (A) Anti‐PD‐L1 binding to human PD‐L1‐Fc as detected by ELISA. 8A1, chimeric anti‐PD‐L1; #77, humanized anti‐PD‐L1; Atezo, atezolizumab (Tecentriq) analogue. (B) Anti‐TIGIT binding to human TIGIT‐Fc as determined by ELISA. 31C3, chimeric anti‐TIGIT; T26 and T87, humanized anti‐TIGIT. (C) Anti‐PD‐L1 antibodies blocked the interaction of PD‐L1 with PD‐1 based on ELISA. (D) Anti‐TIGIT antibodies neutralized the binding of TIGIT to PVR based on ELISA. (E) Human IL‐2 production in the Staphylococcal enterotoxin B (SEB)‐activated peripheral blood mononuclear cells (PBMC) assay in the presence of anti‐PD‐L1 antibodies. (F) Human IFNγ production in primary T cells stimulated with OKT3 and anti‐CD28 in the presence of anti‐TIGIT antibodies. hRS7, a humanized IgG1 mAb against Trop 2, was used as a negative control. (G) MC38‐hPD‐L1 tumour cells in C57BL/6‐HU‐PD‐L1 transgenic mice. Anti‐PD‐L1‐hu#77 was administered i.p. at 1 and 5 mg/kg, and atezolizumab analogue at 5 mg/kg every 3 days. Tumour volume was monitored using electronic callipers every other day. (H) MC38 tumour cells inoculated in C57BL/6‐HU‐TIGIT transgenic mice. Anti‐TIGIT was injected i.p. at 5 mg/kg and tumour size was measured every 3 days. Tumour volumes were presented as mean ± SEM. Statistical significance (*p*‐value) was determined using one‐way or two‐way ANOVA with Tukey's multiple comparison test (*p < .05, **p < .01, ***p < .001)

The biological functions of mAbs were then examined. They all effectively boosted the cytokine release by activated human PBMC evaluated in SEB or anti‐CD3/anti‐CD28 stimulation assay in vitro (Figure 1E,F). Then the antitumor efficacy was investigated in a transgenic mouse model (C57BL/6‐HU‐PD‐L1 or C57BL/6‐HU‐TIGIT), which expressed human PD‐L1 or TIGIT as substitutes for mouse orthologs. Anti‐PD‐L1 antibody potently inhibited the tumour growth of MC38‐hPD‐L1 (Figure 1G; Figure S3) and anti‐TIGIT inhibited MC38 cells (Figure 1H). Anti‐PD‐L1 antibodies also showed potent tumour suppression efficacy in C57BL/6 wild‐type mice (Figure S4).

To obtain a well‐behaved anti‐PD‐L1/TIGIT molecule, eight BsAb antibodies (Bs#1–Bs#8) containing anti‐TIGIT‐huT26 or huT87 in IgG4, and anti‐PD‐L1‐hu#77 in scFv were generated (Figure S5A). Additionally, the sequences of VH and VL in the scFv segment (VH‐linker‐VL and VL‐linker‐VH) with and without an additional disulphide bond mutation were taken into consideration.^8^

We measured the binding kinetics of eight BsAbs by BLI technology on Octet^9^ to both human PD‐L1‐his and human TIGIT‐his, which indicated that the affinities to human PD‐L1‐his (6‐9 nM) (Table 1) slightly decreased compared to humanized mAb (2.66 nM), but affinities to TIGIT were not affected (Figure S1).

**TABLE 1:** KD values and monomer ratios of bispecific antibodies (BsAbs)

	**BsAbs**	**KD (nM)**		**Monomer ratio**		
		**hPD‐L1**	**hTIGIT**	**Native**	**60°C–1 h**	**Decrease**
Bs#1	T26‐77HL‐wt	7.57	1.79	82.2%	70.5%	11.70%
Bs#2	T26‐77LH‐wt	6.12	2.00	83.3%	79.5%	3.80%
Bs#3	T87‐77HL‐wt	6.02	2.37	81.7%	68.6%	13.10%
Bs#4	T87‐77LH‐wt	6.80	1.89	84.6%	81.1%	3.50%
Bs#5	T26‐77HL‐mu	9.03	4.08	87.2%	84.7%	2.50%
Bs#6	T26‐77LH‐mu	7.80	4.21	96.1%	86.1%	10.00%
Bs#7	T87‐77HL‐mu	7.24	3.30	87.9%	83.7%	4.20%
Bs#8	T87‐77LH‐mu	6.16	2.11	95.5%	87.3%	8.20%

**^Notes:^ :**KD values were detected by Octet, and monomer ratios were analysed through SEC‐HPLC.

Abbreviations: HL, VH‐linker‐VL; LH, VL‐linker‐VH; KD, equilibrium dissociation constant; mu, mutant scFv; wt, wild type scFv.

The binding and neutralization assays of eight BsAbs were analyzed by ELISA (Figure 2A–D) and Fluorescence Activated Cell Sorting (FACS) (Figure 2E–H). They all exhibited comparable binding and blocking capacities (Tables S1 and S2). However, Bs#5 to Bs#8 with the extra disulphide bond mutation had higher binding abilities on FACS. Bs#5 had the highest binding activities, with *EC_50_* values of 0.85 nM for HEK293‐hPD‐L1 and 0.55 nM for HEK293‐hTIGIT.

**FIGURE 2:**
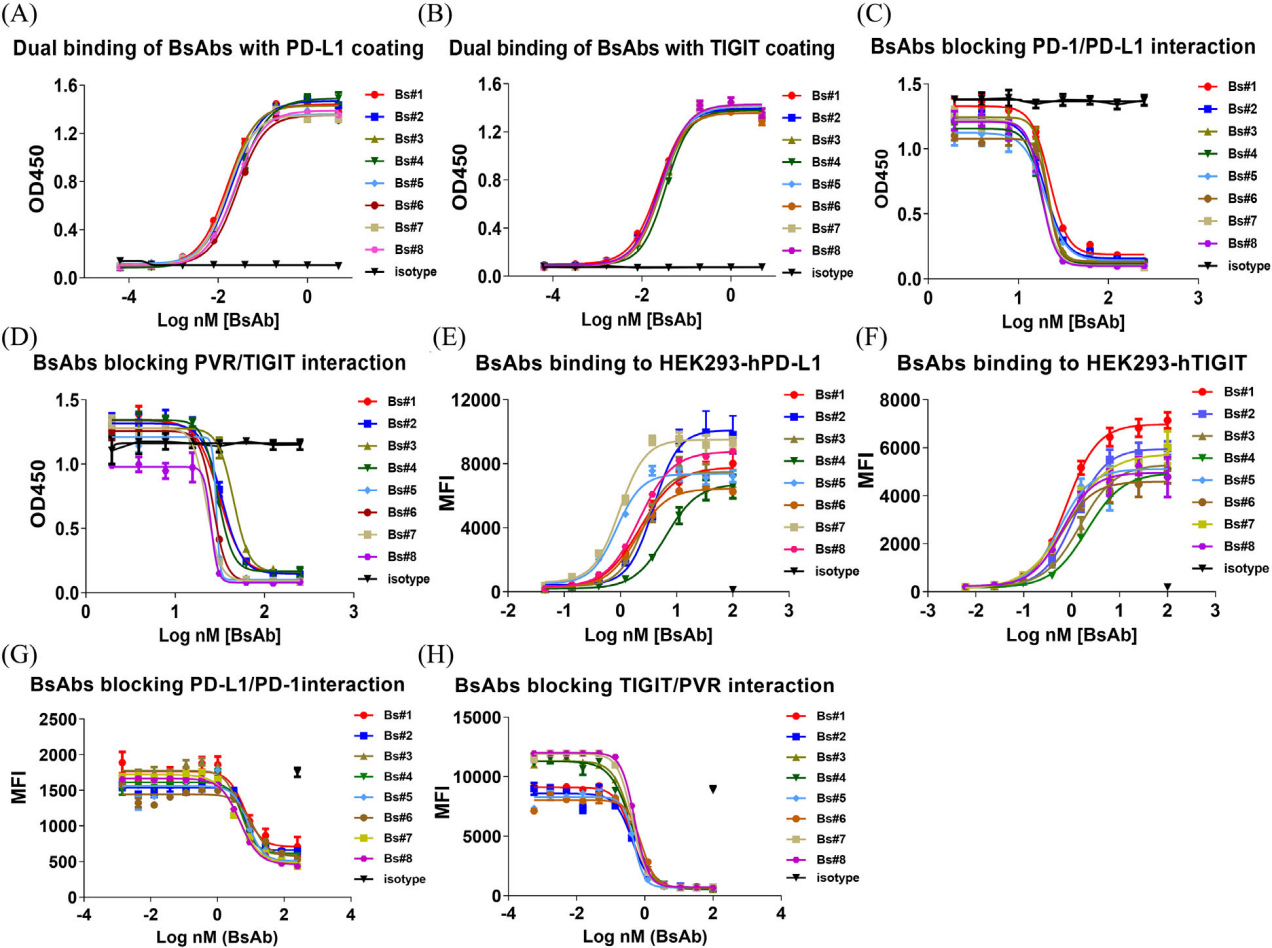
Binding and blocking activities of eight BsAbs determined by ELISA and FACS. (A,B) Dual binding of BsAbs on huPD‐L1‐coating and huTIGIT‐coating ELISA plates. (C,D) Neutralization of PD‐1 binding to PD‐L1 or PVR binding to TIGIT through ELISA. (E,F) BsAbs bound to HEK293‐hPD‐L1 or HEK293‐hTIGIT. (G,H) BsAbs blocked the interaction of HEK293‐hPD‐L1 with hPD‐1 and HEK293‐hTIGIT with hPVR recombinant protein.

Next, the purity and thermostability of BsAbs were evaluated through the HPLC‐SEC method with native (non‐treated) and heat‐treated (60°C in a water bath for 1 h) BsAbs. The monomer ratios in native state and the decrease between the non‐treated and heat‐treated BsAbs revealed that BsAbs with a disulphide bond mutation in scFv (Bs#5 to Bs#8) had better purity (Table 1; Figure S5B). The disulphide bond mutation favoured the VH‐linker‐VL orientation in our BsAbs by comparing Bs#5 (2.5% decrease) to Bs#1 (11.7%), and Bs#6 (10.0% decrease) to Bs#2 (3.8%). Among them, Bs#5 with additional disulphide bond and in VH‐linker‐VL sequence showed better behaviour in purity and thermostability than other BsAbs.

Based on our results, Bs#5 was selected as the BsAb candidate for further evaluation. The affinities and dual binding activity of Bs#5‐hIgG1 (Bs#5.1) were detected by BLI (Figure 3A–D), which exhibited high affinities to both targets and outstanding dual‐binding ability regardless of loading sequences of two antigens. Then, the biological functions of Bs#5.1 were explored in the SEB‐activated PBMC experiment in vitro and double‐gene humanized mouse model (C57BL/6‐HU‐PD‐L1/TIGIT) in vivo. In the PBMC activation assay, Bs#5.1 increased the human IL‐2 secretion sitmualted by SEB to the highest level (Figure 3E–H). In the animal study, Bs#5.1 treatment extended the survival time to the greatest extent and was the only group that showed a significant difference in overall survival compared to the isotype group (Figure 3I,J). However, the BsAb treatment failed to achieve a significant difference in tumour volume due to the great variability within the group. The tumour growth curves were displayed in Figure S6. The variability might arise from the relatively low dose regimen and the heterogenicity of hPD‐L1 expression on MC38‐hPD‐L1 cells (Figure S3).

**FIGURE 3:**
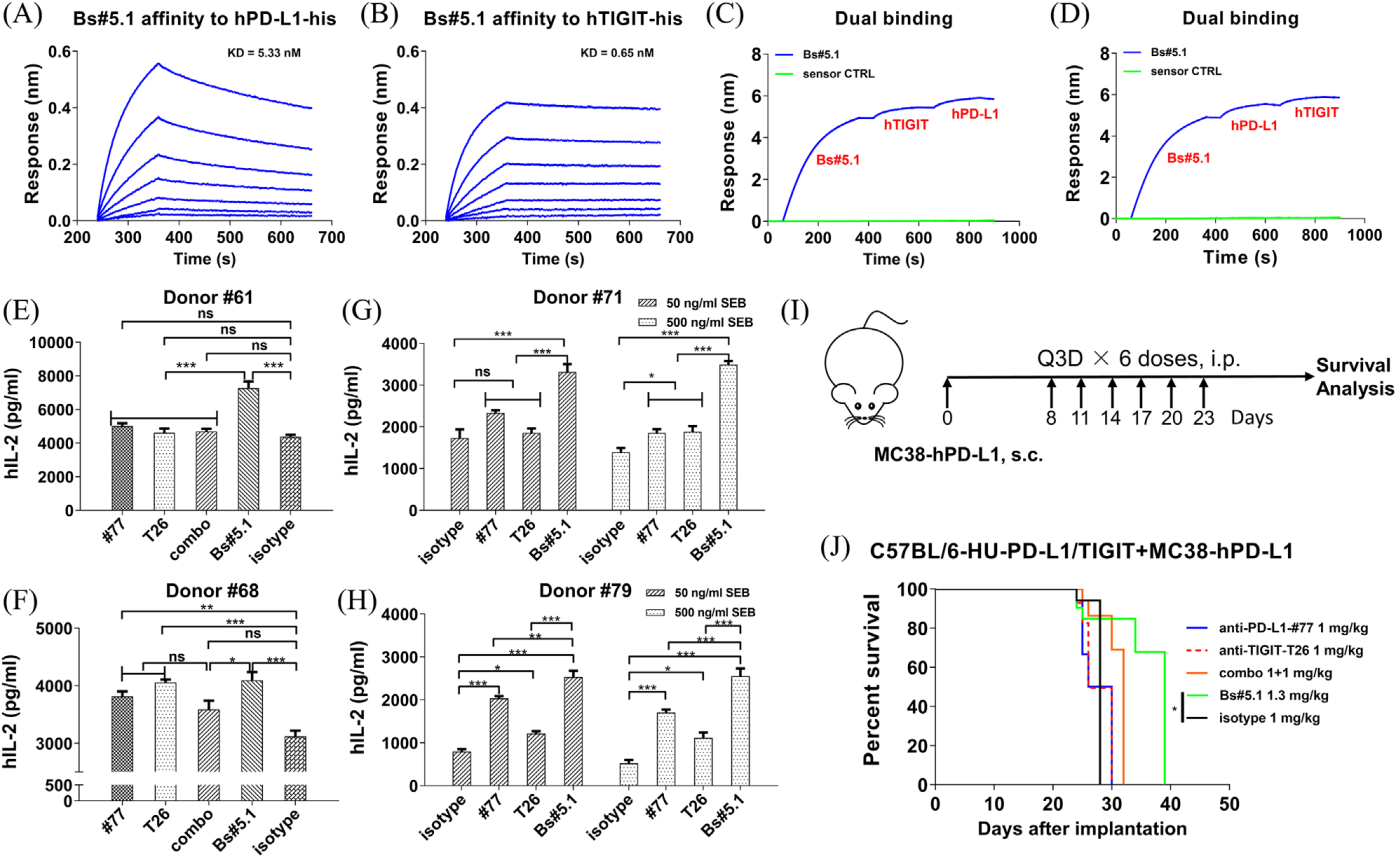
Characterization of BsAb candidates in vitro and in vivo. (A,B) Binding kinetics of Bs#5.1 to hPD‐L1‐his and hTIGIT‐his as determined by BLI using the protein A sensor. (C,D) Dual binding capacity of BsAb on Octet. (E,F,G,H) Bs#5.1 greatly enhanced human IL‐2 production in SEB‐stimulated PBMC assay. Human PBMCs were pre‐incubated with 133.3 nM antibodies at 37°C for 30 min and then stimulated with 1 μg/ml of SEB (E and F) or 50 and 500 ng/ml of SEB (G and H) in 5% CO_2_ incubator for 3 days. Human IL‐2 release in the supernatants was detected by ELISA. Representative graphs were performed in triplicate and data were expressed as mean ± SEM. *P*‐values were determined using one‐way ANOVA with Tukey's multiple comparison test. (I,J) In vivo antitumor activity of Bs#5.1 in MC38‐hPD‐L1‐engrafted C57BL/6‐HU‐PD‐L1/TIGIT transgenic mouse model. (I) Diagram of BsAb animal study. (J) Survival analysis of MC38‐hPD‐L1 tumour‐bearing mice. C57BL/6‐HU‐PD‐L1/TIGIT mice were inoculated with 5 × 10^5^ MC38‐hPD‐L1 tumour cells. Antibodies were administered every 3 days, and tumour volumes were monitored by an electric calliper every other day. Survival analysis was conducted using GraphPad software. *n* = 5 mice per group. (ns, not significant *p* > .05, *p < .05, **p < .01, ***p < .001)

Finally, pharmacokinetics (PK) analysis^10^ of Bs#5.1 was conducted in four female transgenic mice following a single dose injection of 10 mg/kg i.v., which showed similar PK curves by two ELISA methods, indicating that Bs#5.1 was intact and stable in mouse serum and capable of binding both targets (Figure S7).

Currently, several bispecific antibodies targeting PD‐L1/TIGIT (such as HLX301 from Henlius, Inc. and PM1022 from Biotheus Inc.) or PD‐1/TIGIT (IBI321, jointly developed by Eli Lilly and Innovent Biologics, Inc.) have been advanced to Phase I clinical trials with no clinical antitumor efficacies reported yet. The combination therapy with PD‐L1 and TIGIT inhibitors has demonstrated clinical benefits (CITYSCAPE study from Roche) in Phase II clinical trials. However,whether bispecific antibodies targeting PD‐1 (or PD‐L1) and TIGIT have advantage over mAb and combination therapies has not been demonstrated.

Herein, we constructed a BsAb targeting both PD‐L1 and TIGIT in the format of IgG‐scFv and systematically characterized its functions in vitro and in vivo. BsAb induced the highest level of IL‐2 production in primary human T cells when compared to the mAbs and combination. In the transgenic animal model, BsAb increased the overall survival to the greatest extent. Taken together, our data favour the BsAb approach over mAb and combination therapies.

## CONFLICT OF INTEREST

Yi‐Li Chen is an employee of Shanghai Mabstone Biotechnologies, Ltd and Chunhe Wang is employed by both Shanghai Institute of Materia Medica and Dartsbio Pharmaceuticals, Ltd. The remaining authors declare no competing financial interests.

## Supporting information



Supporting Information
