# Supplementary material for: PD‐L1/TIGIT bispecific antibody showed survival advantage in animal model
Source: Clin Transl Med. 2022 May 6;12(5):e754. doi: 10.1002/ctm2.754 (PMC9076010; doi:10.1002/ctm2.754)
Supplement: Supplementary file 1 — Supporting Information [file CTM2-12-e754-s001.pdf]

Supplementary Materials for

PD-L1/TIGIT bispecific antibody showed survival advantage in animal model

Songlin Mu[1,4], Zhijuan Liang[1,4], Yongmei Wang[1,4], Wendi Chu[1,4], Yili Chen[2,3], Qi Wang[1,4], Guifeng Wang[1,4, †], Chunhe Wang[1,2,4, †]

Chunhe Wang (wangc@simm.ac.cn), Guifeng Wang (gfwang@simm.ac.cn).

This PDF file includes:

Figures. S1-S7
Tables. S1-S2

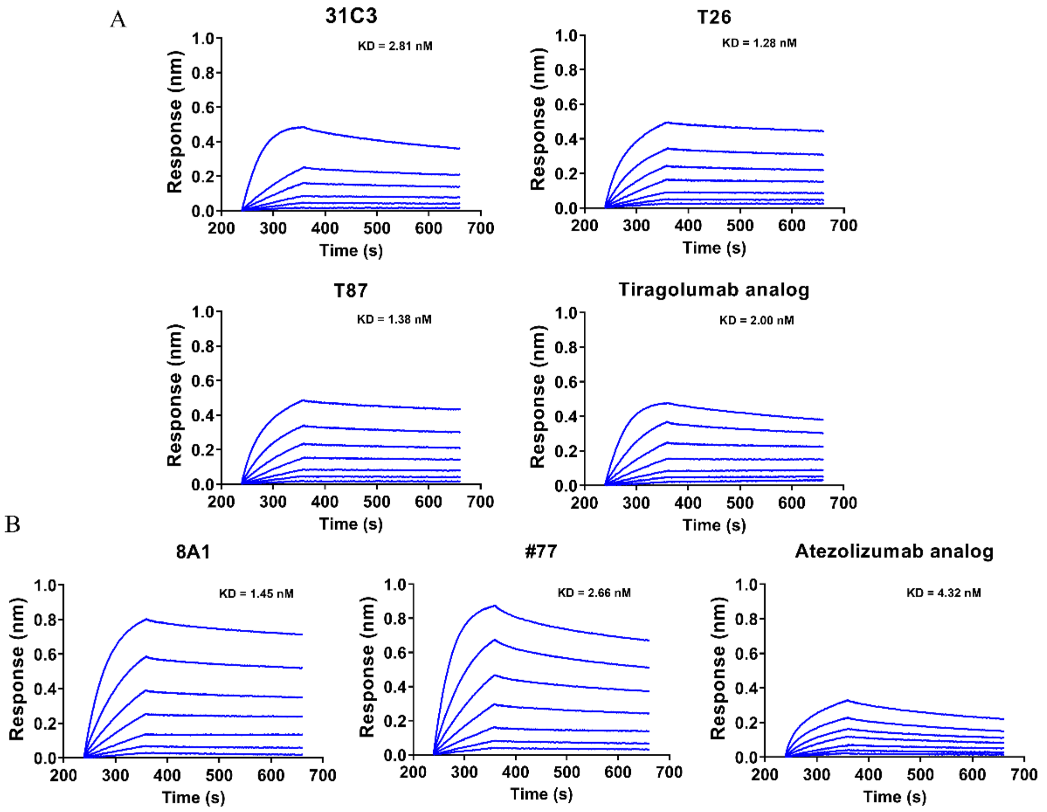

Figure S1. Affinity determination of anti-PD-L1 and anti-TIGIT via biolayer interferometry (BLI). A. Anti-TIGIT and **B.** Anti-PD-L1 binding kinetics by BLI with a ForteBio Octet Red 96 instrument (ForteBio).

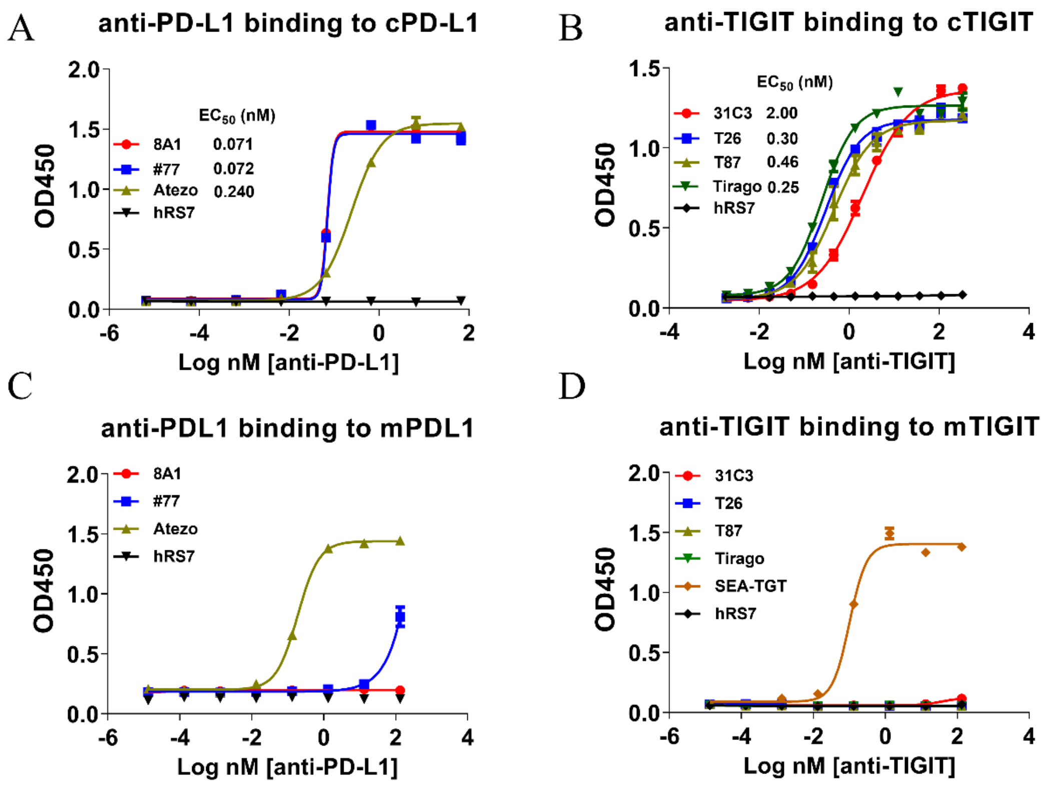

Figure S2. Cross-reactivity of anti-PD-L1 and anti-TIGIT to cynomolgus monkey PD-L1/TIGIT and mouse PD-L1/TIGIT. A. Anti-PD-L1 and **B.** anti-TIGIT binding to cynomolgus monkey PD-L1/TIGIT. **C**. Anti-PD-L1 and **D.** anti-TIGIT binding to mouse PD-L1/TIGIT. SEA-TGT, anti-TIGIT analog from Seagen Inc.

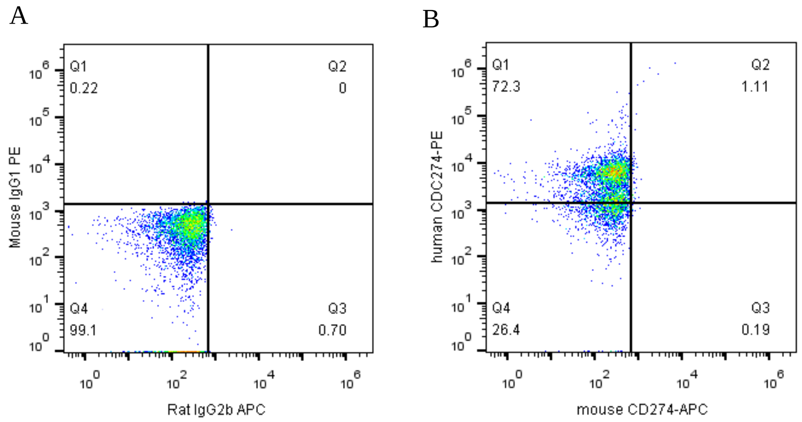

Figure S3. PD-L1 expression on MC38-hPD-L1 cells. MC38-hPD-L1 cells stained with **A.** isotype control and **B.** PE anti-human PD-L1 (BD Pharmingen) and APC anti-mouse PD-L1 (BioLegend).

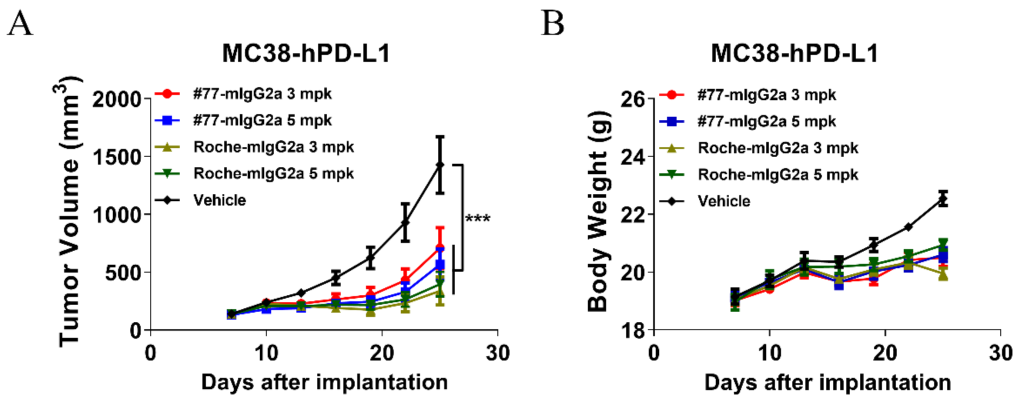

Figure S4. ***In vivo*** **study of anti-PD-L1 in C57BL/6 wild-type mice. A.** Tumor growth of MC38-hPD-L1 tumor-bearing mice. **B.** Body weight during treatment. Tumor volume are expressed as the mean ± SEM. The statistically significant differences were determined by two-way ANOVA with Tukey's multiple comparison test (***$P < 0.001$).

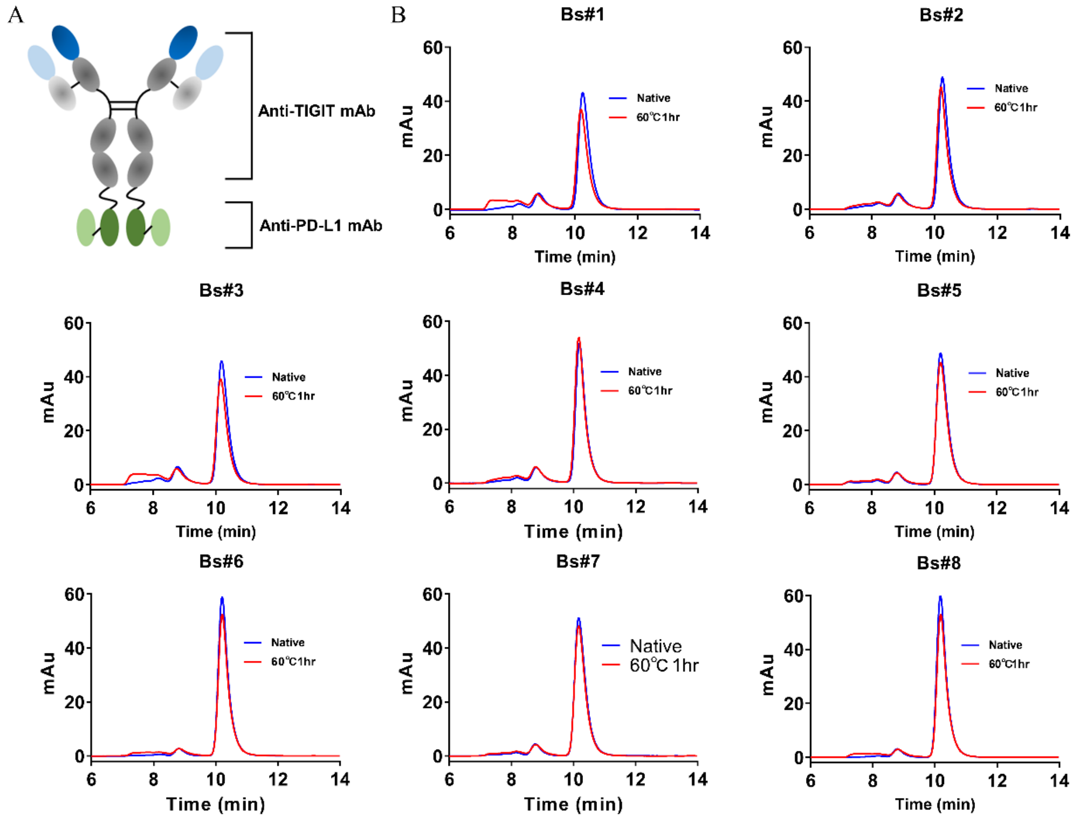

Figure S5. Schematic diagram of anti-PD-L1/TIGIT and thermostability analysis of eight BsAbs through SEC-HPLC. A. The schematic diagram of anti-PD-L1/TIGIT. **B.** SEC-HPLC of eight BsAbs. The native and heat-treated (60 °C for 1 hour in a water bath) BsAb proteins were run on an SEC column at 1 ml/min.

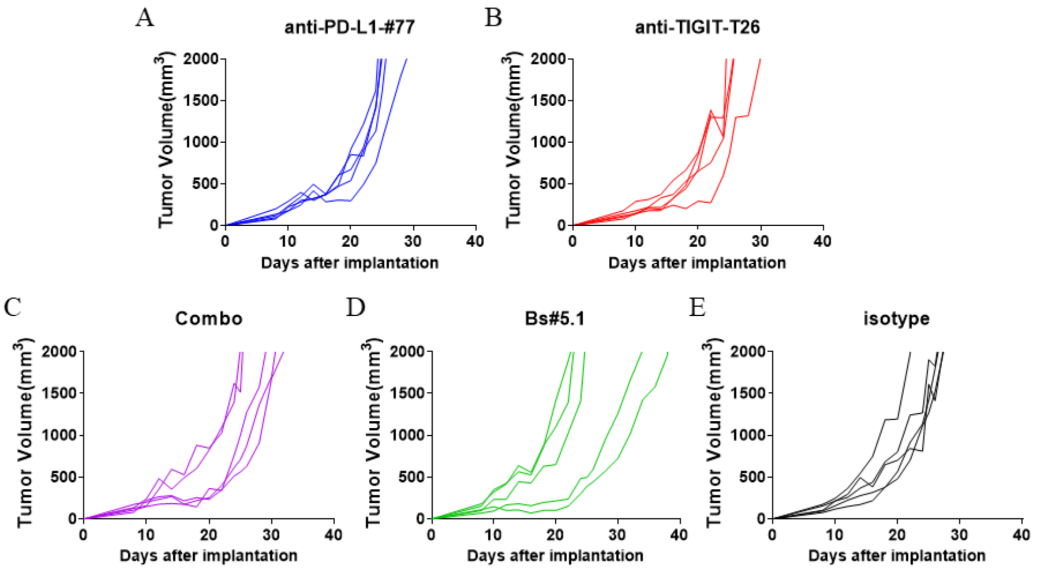

Figure S6. Tumor growth curves of BsAb *in vivo* study. C57BL/6-HU-PD-L1/TIGIT humanized mice were inoculated with 5×10^5 MC38-hPD-L1 tumor cells. Antibodies were administered every 3 days for 6 doses, and tumor volumes were monitored by an electric caliper every other day. Tumor growth curves of **(A)** anti-PD-L1-#77 group **(B)** anti-TIGIT-T26 group **(C)** combo group **(D)** Bs#5.1 group **(E)** isotype group.

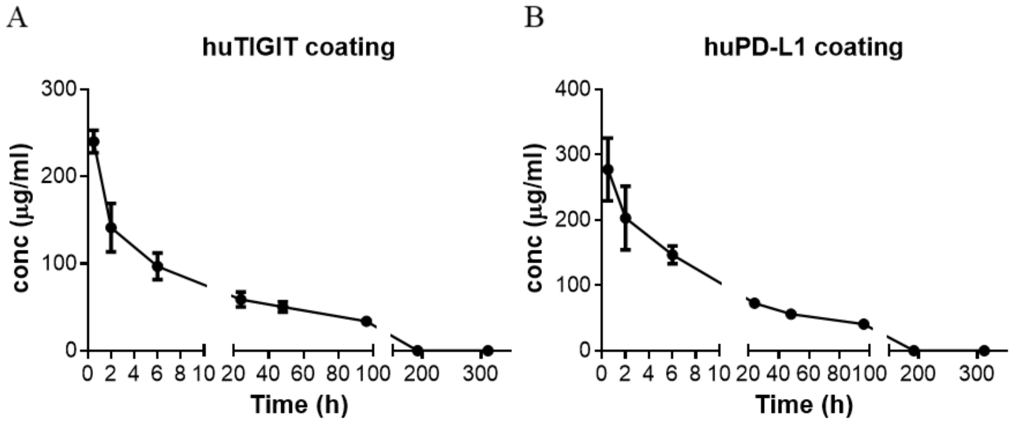

Figure S7. PK analysis of BsAb candidate. A single dose of Bs#5.1 at 10 mg/kg was injected via tail vein into four female C57BL/6J-HU-TIGIT/PD-L1 mice. Serum concentrations of Bs#5.1 at various time points were determined using two sandwich ELISA methods. Bs#5.1 concentration in mouse plasma detected by **A.** hTIGIT coating method and **B.** hPD-L1 coating method.

Table S1. Dual binding and blocking effects of BsAbs on ELISA.

(nM)	Target	Bs#1	Bs#2	Bs#3	Bs#4	Bs#5	Bs#6	Bs#7	Bs#8	isotype
IC_{50}	PD-1 /PD-L1	22.22	19.69	21.08	18.07	19.81	21.62	18.16	18.13	-
	PVR /TIGIT	31.18	33.34	45.02	30.00	28.87	26.64	22.45	25.19	-
EC_{50}	PD-L1 coating	0.016	0.021	0.018	0.027	0.017	0.027	0.017	0.024	-
	TIGIT coating	0.024	0.025	0.027	0.034	0.028	0.026	0.027	0.027	-

† EC_{50}: 50% effective concentration; IC_{50}: 50% inhibitory concentration.

Table S2. Binding and blocking effects of BsAbs on FACS.

(nM)	Targets	Bs#1	Bs#2	Bs#3	Bs#4	Bs#5	Bs#6	Bs#7	Bs#8	isotype
EC_{50}	hPD-L1	2.23	3.87	2.48	6.42	0.85	1.93	0.96	2.17	-
	hTIGIT	0.77	1.13	1.49	2.08	0.55	0.60	0.80	0.65	-
IC_{50}	hPD-L1/hPD-1	6.48	6.86	5.04	6.01	8.36	11.46	4.38	4.72	-
	hTIGIT/hPVR	0.43	0.43	0.43	0.42	0.48	0.79	0.44	0.51	-

† EC_{50}: 50% effective concentration; IC_{50}: 50% inhibitory concentration.
